# Direct single dose mass delabelling of antibiotic allergy in pediatrics

**DOI:** 10.1111/pai.70324

**Published:** 2026-03-12

**Authors:** Sheena Coyne, Aideen Byrne, Amber Gill, Davina Henderson, Kara Tedford, Victoria Mc Donald, Helen O'Connor, Catherine Breen, Aisling Stafford, Maeve Kelleher, David Coghlan, Jonathan Hourihane

**Affiliations:** ^1^ Children's Health Ireland (CHI) Dublin Ireland; ^2^ Royal College of Surgeons Ireland (RCSI) Dublin Ireland; ^3^ Trinity College Dublin (TCD) Dublin Ireland

**Keywords:** antibiotic allergy, delabelling, direct single dose, drug provocation test, mass delabelling, mislabelling, risk stratification

## Abstract

**Background:**

Unsubstantiated antibiotic allergy labels affect between 8% and 25% of the population worldwide. Current risk stratification tools, derived from adult data, are not validated for children. A simplified, multi‐patient protocol with minimal exclusion criteria is required to tackle the scale of this public health issue.

**Methods:**

Patients with possible antibiotic allergy were recruited from the Children's Health Ireland (CHI) allergy waiting list. Exclusion criteria were a serum sickness like reaction (SSLR), severe cutaneous adverse reaction (SCARs), anaphylaxis, or non‐allergic symptoms. No prior allergy testing was performed. Dosing was direct single observed dosing in dedicated mass delabelling clinics, followed by a two‐day home antibiotic course.

**Results:**

Consenting patients (*n* = 162) were seen over 6 clinics with gradually increasing clinic sizes (Range 18 to 62, average 23). One patient only was excluded based on the severity of their index event. Average age was 7 years, *n* = 90/162 (55.6%) were female. Most were avoiding amoxicillin, *n* = 137/162 (84.6%). Negative challenge rates were similar to previous studies, *n* = 150/162 (92.6%), 3 had immediate reactions and 9 delayed (all non‐severe). Patients retrospectively underwent risk stratification according to the 2024 EAACI position paper, high risk *n* = 38/162 (23.5%), intermediate risk *n* = 74/162 (45.7%) and low risk *n* = 50/162 (30.9%). Those deemed high risk were no more likely to have a positive challenge than those deemed low/intermediate risk (*n* = 2/38, 5.3% vs. *n* = 10/124, 8.1%, *p* = .56).

**Conclusion:**

Antibiotic allergy delabelling in pediatrics is low risk and can be done safely in high patient load without prior allergy testing. Current risk stratification tools are not suitable for pediatric‐specific models of care.

**Key message**

This project demonstrates that antibiotic allergy delabelling can be done in large numbers, without prior allergy testing and with minimal exclusion criteria. By using a simplified protocol with minimal patient monitoring, we have shown that delabelling children of antibiotic allergy does not need to be confined to allergists or a hospital setting. The potential impact of involving non‐allergists in delabelling would allow allergists to focus on children with true allergic disease. We have demonstrated to our colleagues poor correlation with recently published EAACI adult‐based risk stratification tools and delabelling outcomes in children. We highlight a need for a pediatric‐specific model of care.

## 1 INTRODUCTION

Antibiotic allergy mislabelling affects between 8% and 25% of the population.^1^ Most labels are given to children before age 3, due to misdiagnosis of viral exanthems.^2^

There is emerging evidence for direct Drug Provocation Tests (DPTs) without prior skin testing (ST) with a 2023 meta‐analysis confirming safety in children with benign delayed reactions.^3^ Two large studies including children with immediate symptoms and a positive DPT found pre‐DPT skin testing to have low sensitivity, 9.1%–20%.^4^, ^5^ Levels of IgE tend to decrease after an index “allergic” event for those with immediate symptoms.^6^ As most immediate reactions are benign and not immunologic in nature, studies have shifted toward direct DPT for non‐severe immediate symptoms but still use graded doses.^7^

The many negative consequences of penicillin allergy labels are well documented, including increased length of stay in hospital, increased cost, increased antimicrobial resistance and increased mortality.^8^, ^9^ A drug provocation test (DPT) or “delabel” can remove 95% of labels and is considered the “gold standard” of drug allergy diagnosis.^3^ Current data on antibiotic allergy in children is not conclusive. As children are much more prone to viral‐induced cutaneous eruptions, adult data on drug reactions cannot be extrapolated to the pediatric population.^10^ Significant discrepancies in the reported chronology of the index event rash and DPT outcome have also been identified in children.^4^ Numerous risk stratification tools for antibiotic allergy have been validated for use in adults, and although modified for children, none have been validated in large international cohorts to date.^11^, ^12^, ^13^

Allergists, both in Ireland and abroad, are unable to meet the current delabelling demand. Prior to this project, the waiting list for DPTs in children in Ireland was up to 4 years. Efforts have been made internationally to support non‐allergists in delabelling, including telehealth.^14^, ^15^, ^16^, ^17^ Data on GP, pharmacist, and parental opinions of antibiotic allergy delabelling as well as the financial burden to families of delayed delabelling is also sparse. A recent Canadian study found parents are in favor of delabelling,^18^ but parental acceptability of delabelling by non‐allergists remains to be determined.

To deal with the scale of the problem, the refutation of unlikely antibiotic allergy in children requires a new approach. Pediatric Allergists are ideally positioned to lead other healthcare professionals in delabelling. Single patient events are not time efficient for hospital clinics. We have previously shown mass challenge efficiency and safety in a higher risk procedure: food challenges.^19^ As there is no universally accepted protocol for DPTs, which can be graded or ungraded,^3^ for the purpose of this study, we will describe the process of administering one non‐graded antibiotic dose with no prior testing as “direct single dose” and the process of removing antibiotic allergy labels in dedicated high volume delabelling clinics as “mass delabelling”. We report here on the planning, implementation and outcomes of mass delabelling of antibiotic allergy in pediatrics using a simplified protocol. This study is the first to our knowledge to examine simultaneous direct single dose delabelling for high numbers of children with both immediate and delayed symptoms using minimal exclusion criteria.

## 2 METHODS

### 2.1 Patient screening

Patients were recruited from the Children's Health Ireland Allergy service waiting list, centrally held for 3 hospitals in Dublin, Ireland. To screen for suitability, all families were telephoned by an allergy registrar using a standardized proforma (Appendix S1). Inclusion criteria were those between 0 and 18 years who had an index event to any oral antibiotic. Patients were excluded if their index event consisted of anaphylaxis,^20^ severe cutaneous adverse reaction (SCARs), serum sickness like reaction (SSLR), non‐allergic symptoms such as headache, or those who had tolerated the implicated antibiotics since their index event.

During screening phone call, information was collected on patient's index event; time since event, morphology/chronology of rash, presence of angioedema or systemic involvement. Information was also gathered on age, sex, co‐morbidities, atopy, medication usage, and family history of antibiotic allergy. Parents were asked how many times (if any) their child was prescribed a second‐line antibiotic since their index event. Second‐line antibiotics were defined as the antibiotics prescribed when the first‐line antibiotic for a specified infection had to be avoided for a suspected allergy. Parents were asked not to bring their child on the delabelling day if they were acutely unwell or had a new rash. A reminder text and phone call were introduced before the last session to improve attendance.

### 2.2 Challenge procedure

On the day of the delabelling, all children were examined by a doctor or allergy nurse specialist to exclude preexisting rash or other conditions. No allergy specific testing was performed. Patients were classified as DNA (did not attend) if they failed to show up or canceled <2 days before the appointment. Patients were given a single age and weight appropriate full dose of the index antibiotic. All antibiotic dosing was as per institutional prescribing guidelines.^21^

One hour post dose, they had a further skin examination before being discharged. To increase the negative predictive value (NPV) and parental confidence in taking the antibiotic again,^22^, ^23^ they were prescribed a 2‐day course of the antibiotic to be completed at home. Before the last challenge, an explanatory letter for families to submit to community pharmacies along with their prescription was introduced. All patients were discharged with the following safety advice: “In the case of reaction, stop taking the antibiotic, take an antihistamine if the rash is itchy and contact the allergy department”. All patients received a follow up call 4 days post delabelling. If delayed symptoms occurred, families were encouraged to send photographic evidence for review by study team.

An immediate reaction was predefined as one occurring within 1 h of the first dose and delayed as >1 h after the first dose and up to 3 days after the last dose. A positive result was classified as one producing objective symptoms such as urticaria, angioedema, new rash, respiratory symptoms, or cardiovascular compromise.^24^

Patients were seen in a hospital day ward with 6 seated bays and one resuscitation bay. Oxygen and resuscitation equipment were available. For sessions 1–5, there were 5–7 rotations of 5 patients per day, allowing for a maximum of 25–35 patients per day. Each patient was allocated 1.5 h. For session 6, we moved to a larger outpatient department with more staff and capacity for 62 patients/day. Patients were seen in 4 clinic rooms and monitored in 2 large waiting rooms; in the case of an immediate reaction, 3 day ward beds were available alongside. For each patient, the time taken to complete delabelling was recorded.

Pre‐post Questionnaire; In order to assess for changes in attitude, parents were asked to complete an anonymous online questionnaire using a QR code before and 1–2 weeks post delabelling. The questionnaires used a 5‐point likert scale (Appendix S1).

### 2.3 Data analysis

A list of antibiotic costs including liquid and tablet formulations was obtained through hospital pharmacy. Cost of the delabelled antibiotic was compared to second line antibiotics. Community doses were based on national prescribing guidelines used by General Practitioners (GPs) and calculated based on current weight of child.^25^ As the EAACI 2024 position paper was published during the course of this study, patients were retrospectively categorized into low, intermediate, and high risk using the detailed history prospectively taken. Data were stored on a REDCAP database (15.0.34) and analyzed through STATA (18.0). Ethical approval was granted through CHI ethics committee in January 2024, REC‐380‐23.

## 3 RESULTS

### 3.1 Delabelling

**FIGURE 1:**
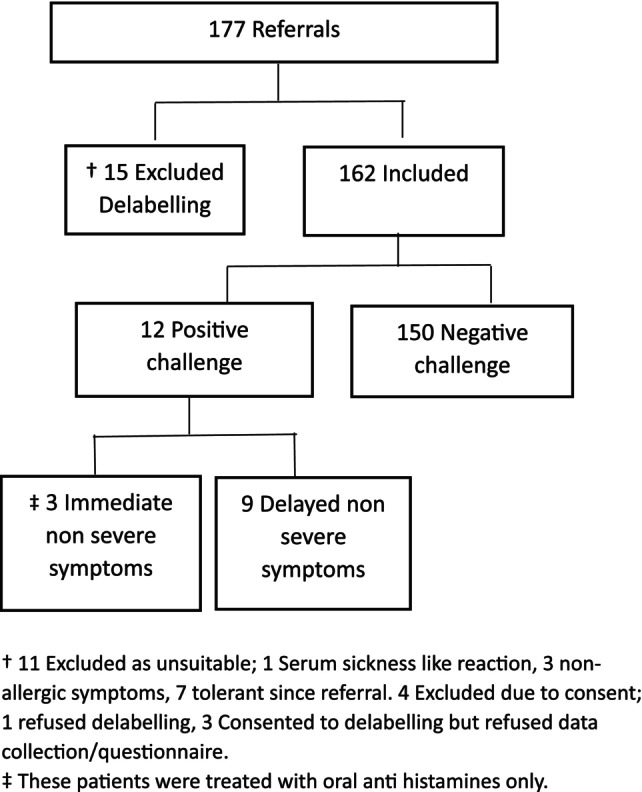
Flow diagram of referrals to pediatric allergy department for antibiotic allergy assessment. ^†^11 Excluded as unsuitable; 1 Serum sickness like reaction, 3 non‐allergic symptoms, 7 tolerant since referral. 4 Excluded due to consent; 1 refused delabelling, 3 Consented to delabelling but refused data collection/questionnaire. ^‡^These patients were treated with oral anti histamines only.

Referral letters were reviewed over 6 months by the allergy department, *n* = 177. Fifteen patients were excluded, 1 patient was excluded on the basis of a SSLR, 3 for non‐allergic symptoms, and 7 for parental reported tolerance to the antibiotic since referral. One patient refused the delabelling, 3 patients consented to the delabelling but not the data collection/questionnaire, Figure 1.

Suitable patients, *n* = 162 were seen over 6 clinics in gradually increasing clinic numbers (16–62 patients per clinic). This reduced the current drug allergy waiting list to 12 months. The mean age was 7 years (SD 4.2, range 6 months–18 years) and *n* = 90/162 (55.6%) were female. A relatively high proportion, *n* = 66/162 (41%) had their index event >3 years ago and had been on the waiting list since then.

The non‐attendance/“(DNA)” rate averaged at *n* = 8 (23%) per clinic with 26/42 (61.9%) of DNAs due to viral illnesses. The DNA rate dropped significantly once a reminder phone call and text were introduced, with no DNAs in session 6. The most frequently implicated antibiotic was amoxicillin (84.6%), Table 1.

**TABLE 1:** Index event antibiotic and indication.

	*N* (% of total)	Negative challenge *N* (%)	Positive challenge *N* (%)
Index event antibiotic			
Amoxicillin	137 (84.6%)	126 (91.9)	11 (8.1)
Co amoxiclav	12 (7.4%)	12 (100)	0
Cefalexin	4 (2.5%)	4 (100)	0
Clarithromycin	4 (2.5%)	4 (100)	0
Flucloxacillin	2 (1.2%)	2 (100)	0
Phenoxymethylpenicillin	3 (1.9%)	2 (66.7)	1 (33.3)
Indication for index event antibiotic			
Tonsillitis	61 (37.7)	58 (95.1)	3 (4.9)
RTI	59 (36.4)	54 (91.5)	5 (8.5)
Ear infection	24 (14.8)	21 (87.5)	3 (12.5)
Cellulitis	6 (3.7)	6 (100)	0
Unknown	7 (4.3)	7 (100)	0
UTI	4 (2.5)	4 (100)	0
Dental	1 (0.6)	0	1 (100)

Abbreviations: RTI, Respiratory Tract Infection; UTI, Urinary Tract Infection.

Overall negative challenge rate for delabelling were high, *n* = 150/162 (92.6%). There were *n* = 3/162 immediate reactions, all mild consisting of hives only appearing within 1 h of first dose and relieved by a second generation antihistamine. Patients were monitored for an extra hour in the allergy department.

A total of *n* = 18/162 (11.1%) patients contacted the department, reporting delayed symptoms (Appendix S1). The history and any available photographic evidence were reviewed by the study team. Two patients were seen in clinic; *n* = 9/18 (50%) were determined to be non‐allergic symptoms and subsequently declared to have passed their delabelling. Outcomes for non‐allergic symptoms were transient heat rash (*n* = 4), eczema (*n* = 2), no objective symptoms (*n* = 1), insect bites (*n* = 1), and coxsackie virus (*n* = 1, confirmed by laboratory testing).

In the case of the other *n* = 9/18 (50%), it was concluded with photographic support that their symptoms were consistent with a delayed drug eruption. One had developed delayed urticaria and mild facial angioedema; all other reactions were mild cutaneous only and managed at home. The sole patient with angioedema had not reported angioedema in her index event.

Excluding the 3 patients with immediate reactions, *n* = 156/159 (98.1%) finished their supervised test dose and observation period within the target time of 1 h. Chronic health conditions requiring ongoing tertiary hospital care were seen in *n* = 21/162 (13%) of children. Due to initial medication refusal, patients with chronic health conditions, in particular neurodevelopmental issues, were more likely to require extra time to complete the delabelling than those without (*n* = 3/21, 14.3% vs. *n* = 0/141, 0%, *p* < .001; OR → ∞ 95% CI lower bound >5.8).

There was no difference in delabelling outcome for sex, age, parent reported child atopy or family history of antibiotic allergy. Those whose index event was to the first dose of an antibiotic course or had angioedema or immediate urticaria in their index event were not more likely to have a positive delabel, Table 2. Those with a more recent index event however, less than a year prior, were more likely to have a positive result than those with an index event >1 year ago (*n* = 6/39, 15.4% vs. *n* = 6/123, 4.9%, *p* = .03, OR 3.5 95% CI (0.87–14.1)), Table 2.

**TABLE 2:** Participant demographics and potential risk factors for delabelling outcomes.

	Total *N* (%)	Negative challenge *N* (%)	Positive challenge *N* (%)	*p* Value	Odds ratio (95% confidence interval)
Participants	162	150 (92.6)	12 (7.4)		
Age					
<5 (or equal to)	69 (42.6)	63 (91.3)	6 (8.7)	.59	
>5	93 (57.4)	87 (93.5)	6 (6.5)		0.72 (.18‐2.85)
Sex					
Female	90 (55.6)	82 (91.1)	8 (8.9)		
Male	72 (44.4)	68 (94.4)	4 (5.6)	.42	0.61 (0.13–2.38)
Time since index event					
<1 year	39 (24.1)	33 (84.6)	6 (15.4)	.03	3.5 (0.87–14.1)
>1 year	123 (75.9)	117 (95.1)	6 (4.9)		
Parent reported child atopy					
Eczema	56 (34.6)	53 (94.6)	3 (5.4)	.47	0.61 (0.1–2.59)
Allergic Rhinitis	43 (26.5)	39 (90.7)	4 (9.3)	.58	1.42 (0.29–6.65)
Food Allergy	21 (13)	19 (90.5)	3 (9.5)	.69	1.38 (0.14–7.23)
Childhood wheeze/asthma	50 (30.9)	47 (94)	3 (6)	.64	0.73 (0.12–3.1)
Any atopy in child^a^	98 (60.5)	91 (92.9)	7 (7.1)	.87	1.5 (0.39–6.05)
Presence of angioedema in index event					
Angioedema	23 (14.2)	21 (91.3)	2 (8.7)	.68	1.23 (0.12–6.38)
No angioedema	139 (85.8)	129 (92.8)	10 (7.2)		
Immediate urticaria in index event					
Immediate urticaria present	28 (17.3)	27 (96.4)	1 (3.6)	.39	0.41 (0.01–3.09)
No immediate Urticaria	134 (82.7)	123 (91.8)	11 (8.2)		
Dose reacted to (if known)					
Reacted first dose	57 (3.3)	54 (94.7)	3 (5.3)	.4	0.56 (0.09–2.39)
Reacted to subsequent dose	100 (63.7)	91 (91)	9 (9)		
Unknown	5				
Family History of antibiotic allergy					
Family History	38 (23.5)	34 (89.5)	4 (10.5)	.47	1.7 (0.35–6.81)
No family History	124 (76.5)	116 (93.5)	8 (6.5)		
Chronic Health conditions^b^					
Chronic health condition	21 (13)	20 (95.2)	1 (4.8)	.62	0.59 (0.01–4.5)
No chronic health condition	141 (87)	130 (92.2)	11 (7.8)		
Risk stratification					
Low	50 (30.9)	48 (96)	2 (4)	^c^	
Intermediate	74 (45.7)	66 (89.2)	8 (10.8)	.27 L v I/H	0.43 (0.04–2.12)
High	38 (23.5)	36 (94.7)	2 (5.3)	.56 H v I/L	0.63 (0.06–3.19)

^a^ Any atopy in a child; Any child whose caregiver reported either eczema, allergic rhinitis, food allergy or childhood wheeze.

^b^ Chronic health condition; Defined by the study team as any health condition (as per International Classification of Diseases 11th Revision) requiring ongoing follow up from a pediatric consultant.

^c^ L, low; I, Intermediate; H, High.

**FIGURE 2:**
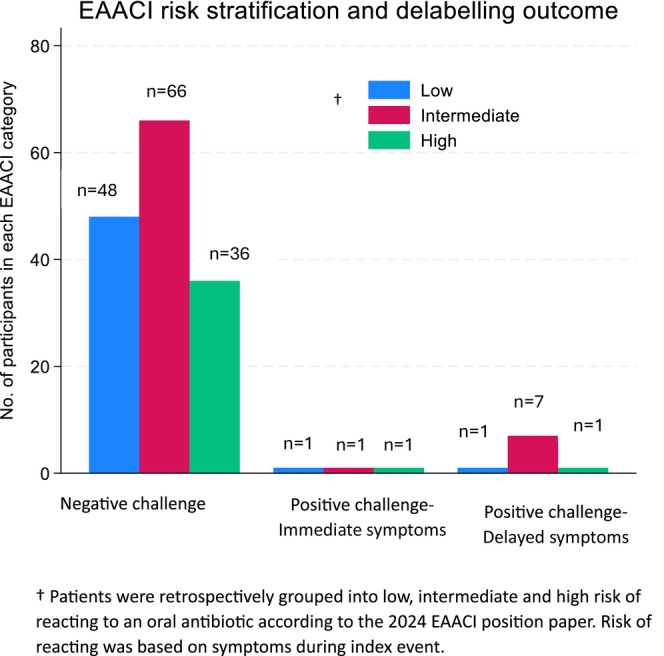
EAACI risk stratification categories (low, intermediate, and high) versus delabelling outcome). ^†^Patients were retrospectively grouped into low, intermediate, and high risk of reacting to an oral antibiotic according to the 2024 EAACI position paper. Risk of reacting was based on symptoms during index event.

Patients retrospectively underwent risk stratification according to the 2024 EAACI position paper and were classified into high risk *n* = 38/162 (23.5%), intermediate risk *n* = 74/162 (45.7%), and low risk *n* = 50/162 (30.9%).^24^ This risk stratification did not correlate with delabelling outcomes. Those deemed high risk were no more likely to have a positive result than those deemed low/intermediate risk (*n* = 2/38, 5.3% vs. *n* = 10/124, 8.1%, *p* = .56 (OR = 0.63; 95% CI, 0.06–3.19)), Table 2. There was equal distribution of risk category for the 3 with immediate reactions post delabelling, Figure 2. Of the 12 that had a positive delabel, just *n* = 4/12 (33.3%) had rash morphology and *n* = 8/12 (66.6%) rash chronology similar to index event.

After mass delabelling sessions 1–4, *n* = 10 queries were recorded from community pharmacists regarding the safety of dispensing the 2 day “at home” component of the delabelling. After the letter for families to submit to community pharmacies was introduced, no calls were recorded at the subsequent mass challenge.

### 3.2 Questionnaire

All 162 parents consented to the pre delabelling questionnaire; due to an IT error, 145 were available for final analysis. We found 99% of those with negative challenges willing to use the implicated antibiotic again. Most patients reported being worried about their child's antibiotic allergy label, *n* = 119/145 (82.1%) and *n* = 74/145 (51%) were worried about the risk of other allergies as a consequence. Almost all, *n* = 42/145 (98%) believed it was important for their child to undergo antibiotic allergy testing.

A total of *n* = 78/162 (48.1%) completed the post delabelling questionnaire with no loss of data. Despite being told that their chances of a reaction were only 5%–10%, *n* = 35/78 (45%) reported having anticipated a reaction would occur. Almost all, *n* = 75/78 (96.2%) found the delabelling process straightforward. Of those who passed, *n* = 73/74 (99%) reported being confident to administer the antibiotic in the future.

The acceptance of community delabelling did not change before and after the delabelling, GP (*n* = 130/145, 89.7% vs. *n* = 67/78, 86%), community pharmacy (*n* = 100/145, 69% vs. *n* = 51/78, 65.4%) and at home via a webcam (*n* = 65/145, 44.8% vs. *n* = 31/78, 39.7%).

### 3.3 Costings

After the index event and before the antibiotic challenge, second line antibiotics had been prescribed to *n* = 128/162 (79%) in total and *n* = 120/150 (80%) of those delabelled (Mean 4.8, SD 4.5, range 1–20). In total 624 alternate antibiotics were prescribed at a cost of €3526.7. As first line antibiotics would have cost €2148.12, €1378.58, or €11.49 per patient would have been saved by families had they been delabelled post index event.

## 4 DISCUSSION

This study highlights antibiotic allergy delabelling in children can be done in high patient load, without prior allergy testing and with minimal exclusion criteria. We have shown that without impacting patient safety and familial satisfaction, it is possible to have a rapid positive impact on hospital waiting lists for drug allergy delabelling.

We have demonstrated poor correlation with current risk stratification tools and delabelling outcomes in children. Popular tools such as PEN‐FAST have not been validated in children.^11^, ^13^, ^26^ PEN‐FAST along with the EAACI/EDNA 2024 position paper are mostly derived from adult data placing considerable weight on urticaria and angioedema within 1 h of antibiotic; this profile was not associated with increased likelihood of reaction in our cohort. In children, viral infections are common triggers for urticaria and angioedema, refuting them as strong indicators of IgE drug allergy.^26^ Delayed onset rashes occur in up to 5% of children treated with beta lactams, the underlying cause of which may involve a viral‐induced polyclonal activation of lymphocytes or differences in drug metabolism.^10^, ^27^ Such is the burden of viral infections in the pediatric population that some studies have incorporated viral testing when performing drug challenges.^28^ We have also confirmed, as in other studies, the limitations in reliability of the index event rash chronology and delabelling outcome.^4^ Due to the timing of the EAACI 2024 position paper, risk classifications were applied retrospectively but using a detailed prospectively taken history; therefore, bias was minimal.

Lack of concordance with adult risk stratification tools could also be explained by age‐related immunological differences between adults and children, with children showing T cell dominant immune profiles and incomplete B cell maturation.^29^ Adults have higher circulating memory B cells reflecting a more adaptive and persistent immune response.^29^ The only significant risk factor identified in our cohort, although with a wide confidence interval, was time since the index event, with most of those reacting having had their index event in the last year. These age‐related immunological differences could explain why the natural history of beta‐lactam allergy in children demonstrates tolerance acquisition for delayed hypersensitivity; our findings support this.^22^, ^30^ Resensitization, although possible, is much less common in children; some adult studies support retesting after negative challenges, especially if the index event was severe.^31^, ^32^ Validated, pediatric‐specific risk stratification tools incorporating the unique features of childhood illness, such as predisposition to rashes, are warranted.

As no validated questionnaire concerning parental acceptability of delabelling existed, we created and used an unvalidated questionnaire. To reduce social desirability bias, responses were anonymous and answers included neutral options. Like previous studies we found parents in favor of delabelling with almost all patients with negative challenges willing to use the challenge antibiotic again, follow up over a longer period is however warranted.^18^ 45% believed their child was going to react during the delabelling; however, parents still overwhelmingly felt their child was safe (92%). One published study found the emergency department not an acceptable place to parents for delabelling. Our study found a higher acceptability of non‐allergist community delabelling (GP, pharmacist); this may reflect preference and trust in community healthcare providers already known to them.^33^ We therefore conclude that tertiary level care for delabelling is not parent driven, and community delabelling may be worth pursuing. Parental acceptance of the delabelling process and willingness to use the implicated antibiotic again are vital for long‐term public health gain.

All of those requiring extra time for delabelling had neurodevelopmental issues and in retrospect were not suitable for the high volume, multipatient mass delabelling model. As no patients in the group without chronic health conditions needed extra time, the odds ratio could not be precisely estimated but indicated a strong association (OR tending to infinity; 95% CI lower limit >5.8). These patients required extra time due to initial medication refusal. Once they were settled in the department, they took the full dose of the medication. These patients may have been better served in an allergy OPD, with a higher staff patient ratio.

This study demonstrated that even over a short time span, there are modest potential cost savings to families when first line antibiotics remain an option. Delabelling early in life ensures that a lifetime of alternative antibiotic choices is avoided both in a community and hospital setting; this likely would result in much greater cost savings for both patients and health services.

Only one child was excluded from this study based on the severity of their index event. In contrast to much of the existing literature which focuses on children with mild or non‐immediate symptoms,^34^, ^35^ we included all those with immediate urticaria and angioedema in our study. As outcomes were in line with international positive/negative challenge rates,^3^ we strengthen our case to include these cases for direct delabelling.

One of the study's strengths is its real world applicability, the design is generalizable to hospital or community settings. Mass delabelling maximizes staff: patient efficiency, moving from 1:1 to 1:6 and clearing clinic space for children with likely genuine allergic conditions. This novel approach, whilst efficient, did not compromise patient safety or acceptance of the process. It does however take additional planning to commit to the mass events, and more clinical space is needed. In real‐world settings, delabelling of 12 patients in 2 h (2 rounds of 6 patients) with 6:1 staff ratio for clinical nurse specialist and allergy registrar may be the optimum staff/patient ratio. Prior to translation to the community setting, staff should ensure a fully stocked resuscitation trolley and have minimum competencies such as Pediatric Life Support (PLS) or equivalent.

Atopy and beta lactam hypersensitivity are not correlated.^36^ Interestingly, our cohort (patients referred for antibiotic allergy assessment) demonstrated a much higher level of eczema (34.6%) than has been reported by Irish population‐based studies.^37^ Acknowledging that in our study the eczema was parent rather than physician reported, it is possible the higher incidence of eczema could be accounted for by a heightened awareness of allergy in the atopic cohort and an eagerness for drug allergy referral or a patient phenotype more prone to skin rashes contributing to their index event.

There is a lack of a universally accepted protocol on prolonged versus single dose drug challenges; current guidelines recommend balancing safety, risk, and resources.^24^ The initial rationale for the 2‐day antibiotic course in this study was to increase the NPV and parental confidence in giving the dose at home again whilst balancing the theoretical risk of prolonged antibiotic exposure.^22^, ^23^ However, after receiving calls from families that community pharmacists were questioning the course, it was evident that familial confidence in the process was affected. This finding would refute the current argument for prolonged courses. Delayed reactions in this study, like others, were generally mild.^38^ The potential benefit of increasing the NPV by a small amount may not justify the added antimicrobial exposure and impracticalities of the prolonged course. As there is also emerging evidence that the initial dose can produce symptoms up to 3 days post ingestion,^39^ a simple, single dose delabel is both practical and likely sufficient to produce a positive result.

## 5 CONCLUSION

Antibiotic allergy delabelling in children is low risk and can be performed in high patient load in the ambulatory setting. Children are immunologically distinct from adults; our data shows poor correlation of index event symptoms and delabelling outcome, current risk stratification tools therefore do not work for pediatric specific models of care, long term safety data is however warranted.

Capacity of specialized allergy care is increasingly limited. Considering the lack of indication for and poor performance of allergy testing, the safety of delabelling and the scale of the public health issue, a simplified high‐volume pathway like this one is required. This model could be delivered by non‐allergists and even adapted to the community, a model that families report a willingness to engage with.

## AUTHOR CONTRIBUTIONS

**Sheena Coyne:** Conceptualization; methodology; software; data curation; investigation; formal analysis; project administration; visualization; funding acquisition; writing – original draft; writing – review and editing. **Aideen Byrne:** Conceptualization; methodology; data curation; supervision; resources; writing – review and editing; investigation; formal analysis; project administration. **Amber Gill:** Project administration; resources; conceptualization. **Davina Henderson:** Project administration. **Kara Tedford:** Resources; project administration. **Victoria Mc Donald:** Conceptualization; project administration; resources. **Helen O'Connor:** Conceptualization; project administration; resources. **Catherine Breen:** Project administration. **Aisling Stafford:** Project administration. **Maeve Kelleher:** Project administration; writing – review and editing. **David Coghlan:** Writing – review and editing; conceptualization; project administration. **Jonathan Hourihane:** Conceptualization; methodology; data curation; supervision; project administration; writing – review and editing; formal analysis; visualization.

## FUNDING INFORMATION

This project was in receipt of a training grant from Children's Health Foundation. The training grant partially covered MD fees of the first author, the rest was covered by the affiliated University, Royal College of Surgeons Ireland (RCSI), Dublin Ireland. There was no other financial support.

## CONFLICT OF INTEREST STATEMENT

There are no conflicts of interest.

## Supporting information


Appendix S1.
