# Supplementary material for: Direct single dose mass delabelling of antibiotic allergy in pediatrics
Source: Pediatr Allergy Immunol. 2026 Mar 12;37(3):e70324. doi: 10.1111/pai.70324 (PMC12982911; doi:10.1111/pai.70324)
Supplement: Supplementary file 1 — Appendix S1. [file PAI-37-e70324-s001.docx]

Supplemental Figure 1; Delabelling patient workflow


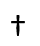

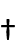

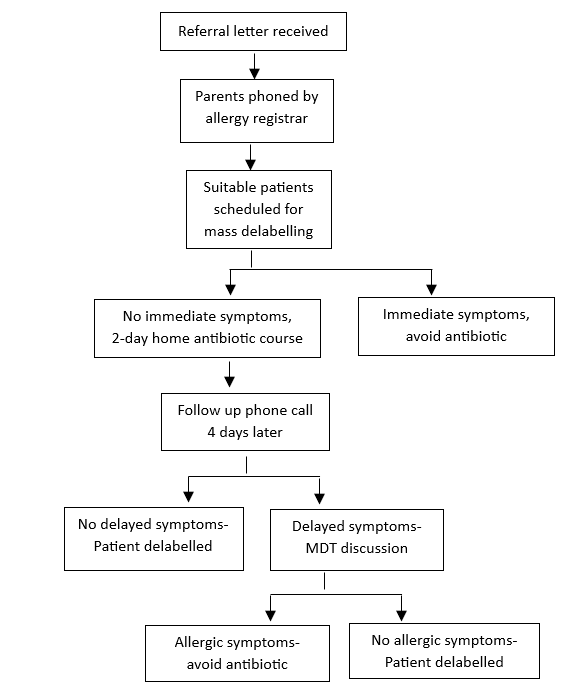


Abbreviations; † MDT; Multidisciplinary Team.

Letters with challenge outcome were sent to the General Practitioners of all patients


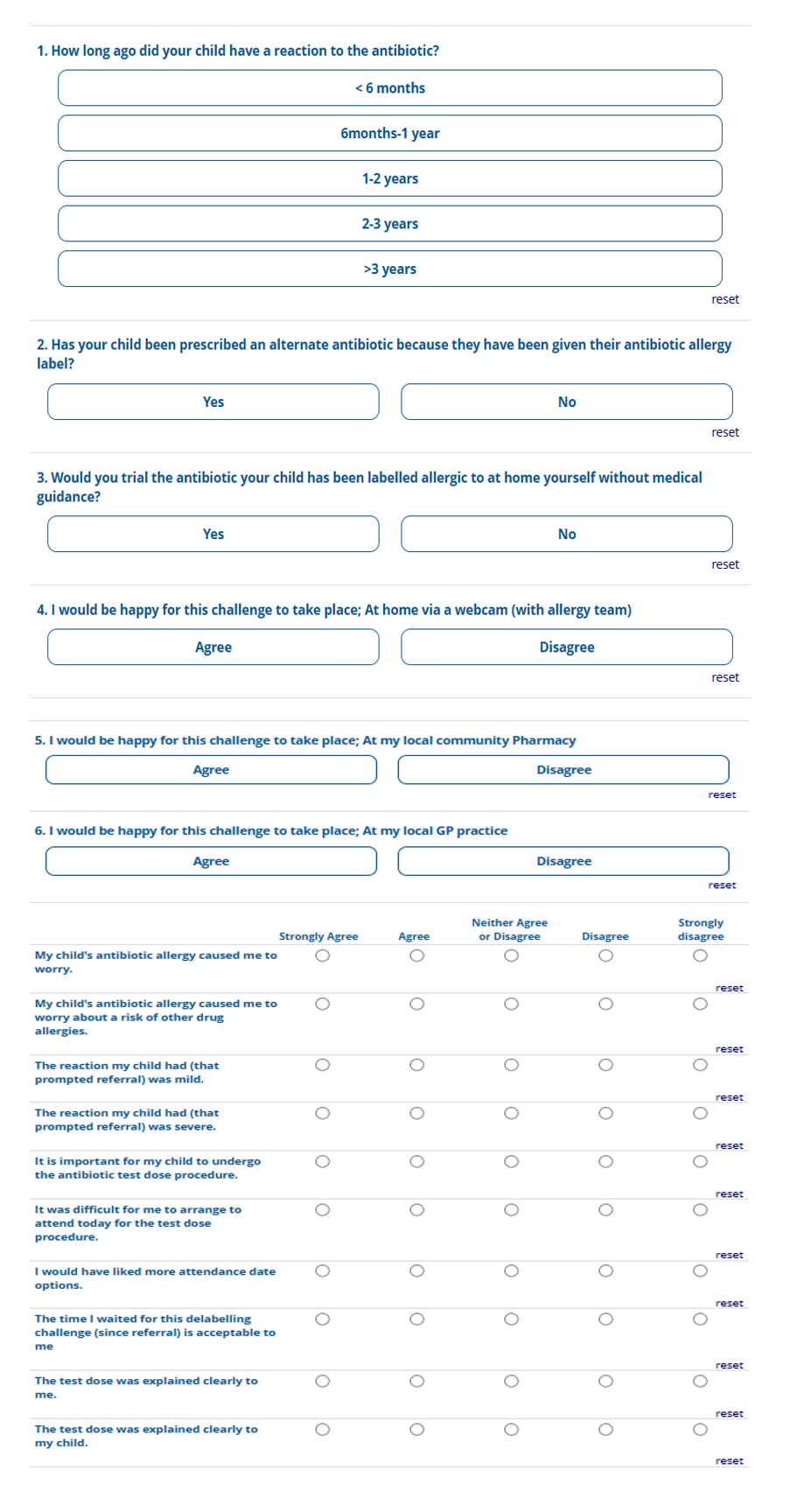


Questionnaire was filled out electronically on caregiver’s electronic in hospital immediately prior to the delabelling procedure. Caregivers accessed the questionnaire via a QR code using their own electronic device. To ensure correct usage, a member of the allergy team helped the caregiver with the first 2 questions. Answers were anonymous.

Supplemental Figure 2; Pre-Challenge care giver questionnaire


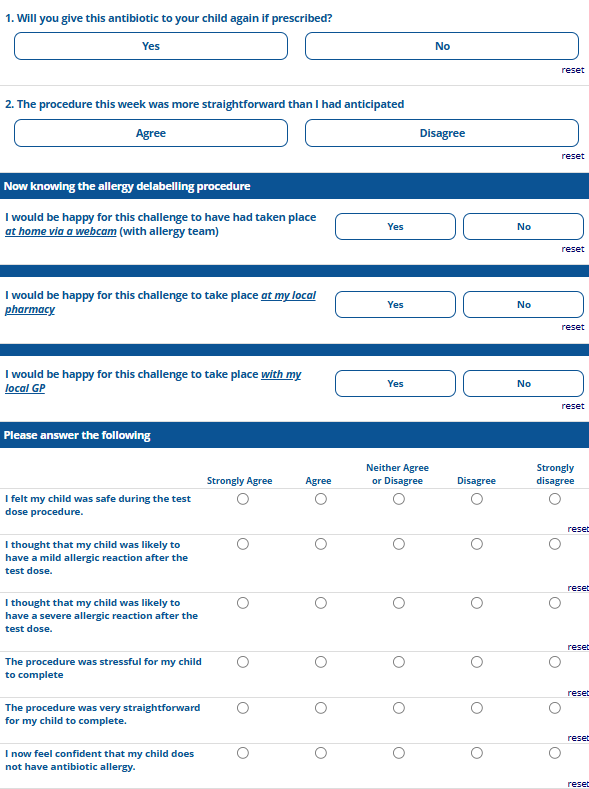


Supplemental Figure 3; Post-Challenge care giver questionnaire

Questionnaire was filled out at home one week after delabelling procedure. Caregivers accessed the questionnaire via a QR code using their own electronic device. Answers were anonymous.

Patient data was filled out using the above proforma and then transferred to REDCAP by the study team. All data was entered using a patient specific study number.

Supplemental Figure 4; Proforma used during mass delabelling sessions


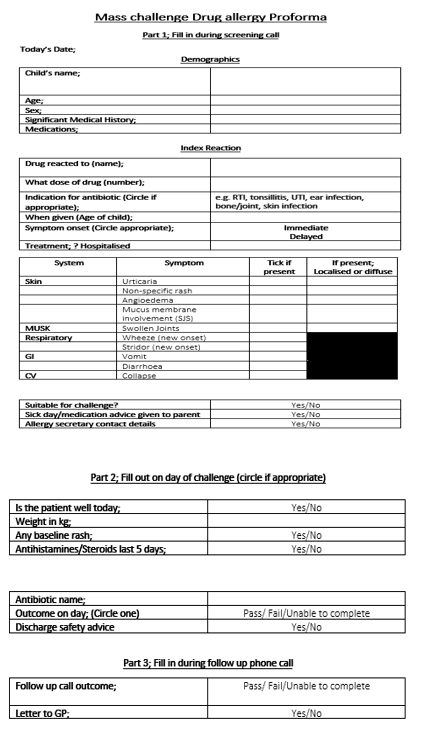


Supplemental Table 1; Patients discussed at Multidisciplinary Team Meeting (MDT) post delabelling

| **Antibiotic** | **Index event symptoms** | **Delabel**  **Symptom onset** | **Symptoms post delabel** | **Symptom duration** | **Comparison with index event (positive challenges only)** | **Outcome of MDT discussion**  **(Negative challenges only)** | **Challenge result** |
| --- | --- | --- | --- | --- | --- | --- | --- |
| Amoxicillin | Urticaria | Day 2 | Pinprick rash | <1 hour | NA | † Heat rash | Negative |
| Co-amoxiclav | Widespread maculopapular rash | Day 2 | Pinprick rash | <1 hour | NA | † Heat rash | Negative |
| Amoxicillin | Immediate Urticaria | Day 2 | Maculopapular rash | 3-4 days | Morphology; no  Chronology; No |  | Positive  Delayed |
| Amoxicillin | Maculopapular rash | Day 2 | Pinprick rash | <1 hour | NA | † Heat rash | Negative |
| Amoxicillin | Immediate non-specific rash | Day 1;  8 hours post first dose | Progressive widespread non-specific rash | 3 days | Morphology; yes  Chronology; no |  | Positive  Delayed |
| Amoxicillin | Delayed urticaria | Day 2 | Urticaria | Few hours | Morphology yes  Chronology yes |  | Positive  Delayed |
| Amoxicillin | Delayed urticaria | Day 2 | Widespread  maculopapular rash | 2 days | Morphology; no  Chronology; yes |  | Positive  Delayed |
| Amoxicillin | Delayed urticaria | Day 2 | Non-specific rash | Unsure | Morphology; no  Chronology; yes |  | Positive  Delayed |
| Amoxicillin | Immediate urticaria | Day 1 | Felt itchy that evening only | <1 hour | NA | No objective symptoms | Negative |
| Amoxicillin | Delayed urticaria | Day 2 | Pinprick rash | <1 hour | NA | † Heat rash | Negative |
| Amoxicillin | Delayed non-specific rash | Day 3 | Urticaria | Unsure | Morphology; no  Chronology; yes |  | Positive  Delayed |
| Amoxicillin | Delayed non-specific rash and angioedema | Day 2 | Delayed rash only | 3-4 days | Morphology; no  Chronology; yes |  | Positive  Delayed |
| Amoxicillin | Delayed non-specific rash and angioedema | Day 2 | 2 fixed papules | 3-4 days | NA | ‡ Insect bites | Negative |
| Amoxicillin | Delayed non-specific rash | Day 3 | Erythema flexural surfaces | Resolved with steroid use | NA | Eczema | Negative |
| Amoxicillin | Delayed non-specific rash and angioedema | Immediate | Urticaria | Resolved with antihistamine | Morphology; no  Chronology; no |  | Positive  Immediate |
| Amoxicillin | Delayed urticaria | Day 2 | Facial angioedema and non-urticarial rash | Resolved with antihistamine | Morphology; no  Chronology; yes |  | Positive  Delayed |
| Amoxicillin | Delayed non-specific rash | Day 3 | Blisters hands, feet, mouth | 4-5 days | NA | § Enterovirus | Negative |
| Phenoxymethylpenicillin | Immediate non-specific rash | Immediate | Urticaria | Resolved with antihistamine | Morphology; no  Chronology; yes |  | Positive  Immediate |
| Amoxicillin | Delayed non-specific rash | Day 2 | Maculopapular rash, progressed | 3 days | Morphology; yes  Chronology; yes |  | Positive  Delayed |
| Amoxicillin | Delayed non-specific rash | Immediate | Urticaria | Resolved with antihistamine | Morphology; no  Chronology; no |  | Positive  Immediate |
| Amoxicillin | Immediate urticaria | Day 2 | Erythema | <1 hour | NA | Eczema | Negative |

† Heat Rash; Diagnosed over phone based on history and photographic support. Continued 2 day course of amoxicillin with no progression.

‡ Insect bites; Diagnosed over phone based on history and photographic support . Continued 2 day course of amoxicillin with no progression.

§ Diagnosed in person, confirmed by laboratory swab, positive for enterovirus.
